# Facilitation of adhesion and spreading of endothelial cells on silicone oxide-coated dacron material by microwave-excited low-pressure plasma

**DOI:** 10.1515/iss-2021-0027

**Published:** 2022-01-14

**Authors:** Daniel J. Tilkorn, Heiko Sorg, Agnes Sanders, Manfred Köller, Peter Awakowicz, Jörg Hauser

**Affiliations:** Department of Plastic, Reconstructive and Aesthetic Surgery, Handsurgery, Alfried Krupp Krankenhaus Essen, Essen, Germany; Department of Health, University of Witten/Herdecke, Witten, Germany; Department of Plastic, Reconstructive and Aesthetic Surgery, Klinikum Westfalen, Dortmund, Germany; Surgical Research, Bergmannsheil University Hospital, Ruhr-University Bochum, Bochum, Germany; Institute for Plasma Technology, Ruhr-University Bochum, Bochum, Germany

**Keywords:** low-pressure plasma, polyethylene terephthalate (PET), silicone, vascular prosthesis

## Abstract

**Objectives:**

Autologous transplants are still the means of choice for bypass surgery. In addition to good tolerability, there is a reduced thrombogenicity and fewer neointima hyperplasia compared to artificial materials. However, since viable transplants are limited, attempts are being made to improve existing artificial vascular prosthesis material. Next to the reduction of thrombogenicity, a rapid endothelialization of the vascular graft should reduce intimal hyperplasia and thus prevent stenoses. The effect of newly developed silicon oxide coatings on the growth of endothelial cells was therefore the goal of this work in a cell culture study.

**Methods:**

A woven, uncoated polyethylene terephthalate (PET) vessel prosthesis was used. The coating process was carried out in a low-pressure plasma reactor in a multi-step process. After preparation of the vacuum chamber hexamethyldisiloxane (HDMSO) with oxygen was evaporated using argon plasma. By this an approx. 1 nm thin adhesion promoter layer was separated from plasma and HMDSO. The silicone oxide barrier layer was applied to the PET vessel samples. The carbon content of the layer could be selectively altered by changing the HMDSO oxygen flow ratio, resulting in coatings of 100 nm, 500 nm, and 1,000 nm. In addition, two different oxygen-to-HMDSO ratios were used. To achieve a carbon coating as low as possible, the ratio was set to 200:1. A carbon-rich layer was obtained with the 1:1 setting. The various coatings were then examined for their surface texture by scanning electron microscopy (SEM) as well as by cell culture experiments for cell viability and growth using EA.hy 926 cells.

**Results:**

SEM showed no changes in the surface morphology; however a layer thickness of 1,000 nm showed peeled off coating areas. Alamar blue assays showed a significantly higher metabolic activity (p=0.026) for the coating 500 nm, ratio 200:1 compared to untreated control samples and a significantly lower metabolic activity (p=0.037) of the coating 500 nm, ratio 1:1 compared to the coating 500 nm, ratio 200:1. This underlines the apparent tendency of the 1:1 coating to inhibit the metabolic activity of the cells, while the 200:1 coating increases the activity. Fluorescence microscopy after calcein acetoxymethyl ester (AM) staining showed no significant difference between the different coatings and the uncoated PET material. However, a tendency of the increased surface growth on the coating 500 nm, ratio 200:1, is shown. The coatings with the ratio 1:1 tend to be less densely covered.

**Conclusions:**

The results of this work indicate a great potential in the silicon coating of vascular prosthesis material. The plasma coating can be carried out easy and gently. Cell culture experiments demonstrated a tendency towards better growth of the cells on the 200:1 ratio coating and a poorer growth on the carbon-rich coating 1:1 compared to the uncoated material. The coating with silicon oxide with a thickness of 500 nm and an oxygen-HMDSO ratio of 200:1, a particularly low-carbon layer, appears to be a coating, which should therefore be further investigated for its effects on thrombogenicity and intimal hyperplasia.

## Introduction

Due to the demographic change, the incidence of cardiovascular diseases such as aneurysms and peripheral occlusive diseases has increased in recent years [1]. Autologous transplants are still the material of choice for bypass surgery. In addition to good tolerability, there is a reduced thrombogenicity and fewer neointima hyperplasia compared to artificial materials [2, 3]. In the case of small vessels artificial prosthesis material leads to increased rates of stenosis. Therefore, and if possible, the saphenous vein must be used [4, 5]. However, since autologous transplants are limited, the perfect vascular prosthesis material is still being developed. One of the main aims in vascular prosthesis research is therefore to develop an ideal vascular prosthesis claiming the possibility of sterilization, storage and rapid availability, no or at least low cytotoxicity, good handling in surgical processing, no induction of external body complaints, no foreign body reaction, low thrombogenicity, variability of caliber; wall thickness; and length, and stability with elasticity and porosity with sufficient tightness [6]. Various surface coatings, for example, attempt to improve artificial vascular prosthesis material [7], [8], [9], [10]. Specifically, the direct and fastest possible cell colonization on the different coatings with endothelial cells was investigated in this context by many groups so far [8, 9, 11], [12], [13]. In addition to the reduction of thrombogenicity, a fast endothelialization of the vascular prosthesis should reduce intimal hyperplasia and thus prevent stenoses.

Until today it has not been possible to develop a vascular prosthesis that meets all the above-mentioned requirements. The present work is therefore dedicated to a novel silicon oxide coating of a polyethylene terephthalate (PET) prosthesis by microwave-excited low-pressure plasmas. Since an improvement in the endothelium-dependent vasodilatation could be demonstrated in the literature during the investigation of silicon oxide-coated stents and the endothelium in healthy vessels plays an important role in the inhibition of smooth muscle cells and reduces the thrombogenicity, it is now examined whether the silicone oxide coating has an effect on endothelial cell growth. In this case, the coating process allows the variation of the coating thickness and the coating composition. In addition, scanning electron micrographs of the coatings should show superficial changes that may occur. Changes in endothelial cell growth are to be demonstrated by experiments in cell cultures. By means of fluorescence photometry and fluorescence microscopy, differences in the growth behavior of the cells and their viability with respect to the different test materials are to be determined.

## Materials and methods

The experimental part of the present work involves the testing of four different silicone oxide material coatings compared to untreated material.

### Vascular prosthesis material

The PET vessel prosthesis material used was woven, uncoated material from Vascutek (VASCUTEK Deutschland GmbH, Hamburg, Germany). The material has not been reinforced or preformed. The porosity is indicated at 358 mL/min/cm^2^ at 120 mmHg. The wall thickness is 0.6 mm. The material was punched in circles of 1.5 cm diameter. On the extraluminal side, the material had a marking strip for position control. After punching, the individual prosthesis material was steam sterilized (Systec DX-23, Systec GmbH, Wettenberg, Germany).

### Coating of the PET fabrics

The coating process was carried out in a low-pressure plasma reactor (Department of General Electrical Engineering and Plasma Technology, Ruhr-University Bochum, Bochum, Germany). The coating parameters were maintained and controlled by the software LabView (National Instruments, Austin, USA). The coating of the PET vessel prosthesis material pieces took place in a vacuum chamber with a volume of 6 × 10^−3^ m^3^. The coating included a three-step process. The basic pressure was 1 Pa. For the coating, hexamethyldisiloxane (HMDSO) was first evaporated and passed into the chamber with oxygen. Here, microwaves (f-2.45 GHz) with a maximum power of P=4 kW were generated. The homogeneous coating along the bottle wall resulted in a pulse interval in which a gas exchange took place before a new plasma pulse ignited. First, a pre-run was performed with a PET bottle for cleaning and sterilizing the bottle. The bottom of the bottle was removed beforehand, and a double-sided adhesive tape was placed circularly on the same level. Initially, only the first step of the coating process was carried out using argon plasma. After removal of the sterilized bottle, the sterile PET samples were attached to the extraluminal side to the previously applied adhesive film. During the subsequent coating step, firstly the activation and purification of the plastic surface by means of argon plasma was carried out. Secondly, an approx. 1 nm thin adhesion promoter layer was separated from plasma and HMDSO. Thirdly, the silicon oxide barrier layer was applied. In this case, the carbon content of the layer could be selectively altered by changing the HMDSO oxygen flow ratio. The higher the ratio of oxygen-to-HMDSO, the less carbonaceous fragments were found in the layers. The coating thickness was initially set at 100 nm, 500 nm and 1,000 nm. For this purpose, the various coating thicknesses were achieved by successively switching the coating process. A cooling time of 5 min was maintained between the coating processes. In addition, two different oxygen-to-HMDSO ratios were used. In order to achieve a carbon coating as low as possible, the ratio was set to 200:1. A carbon-rich layer was obtained with the 1:1 setting. The various coatings were then examined for their surface texture by scanning electron microscopy (SEM).

### Scanning electron microscope

**Figure 1:**
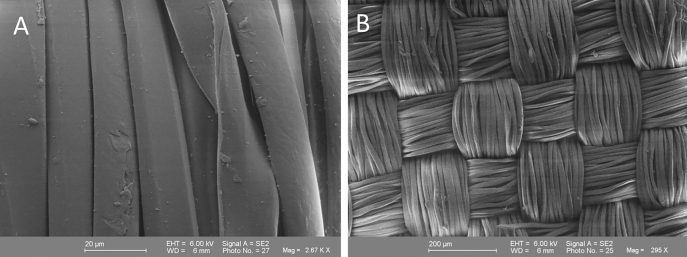
Scanning electron micrographs of an uncoated polyethylene terephthalate (PET) vascular prosthesis (A) and lower magnification of a woven uncoated PET prosthesis material (B) under the scanning electron microscope.

The SEM was carried out at the SEM center of the Ruhr-University Bochum at the Institute of Geology, Mineralogy, and Geophysics (Gemini 1530, LEO, Oberkochen, Germany). The different PET fabrics have been treated first since the SEM can only represent conducting surfaces. First, the PET fabrics must be completely anhydrous since the examination takes place in a vacuum. In addition, they were vaporized with a metal film, in our case gold, which makes the surface conductive. In the present work, the preparations were coated with the Edwards Sputter Coater S150 B (Edwards, West Sussex, United Kingdom) with gold. Uncoated material was used as a control (Figure 1).

### Cell culture experiments

For cell culture experiments the human vascular endothelial cell line EA.hy926 was used. Five different passages of the cells were used in the experimental series (n=5). In carrying out cell culture experiments, attention was paid to sterile conditions. Until the EA.hy 926 cells were used, they were stored in liquid nitrogen. Initially, a cell culture medium was prepared. For this, 4 mL of inactivated fetal calf serum (FCS; Invitrogen, Karlsruhe, Germany) were thawed and filled to 40 mL with RPMI1640 (Invitrogen). Fifteen milliliters of the cell culture medium were filled into a cell culture bottle (BD Falcon, Heidelberg, Germany). In a 15 mL Falcon tube (BD Falcon, Heidelberg) 10 mL of the cell culture medium was filled. Both were preheated for 30 min at 37 °C in an incubator (HERAcell, Kendro Heraeus, Hanau, Germany) in a saturated 5% CO_2_ atmosphere. After removal of the frozen cells from the nitrogen tank, the cells were rapidly thawed by immersing the cyrus tube (2 mL, Nalgene/NUNC GmbH, Wiesbaden, Germany) into a water bath (Köttermann 3042, Köttermann GmbH & Co. KG, Uetze/Hänigsen, Germany). Care was taken to limit the thawing time to a maximum of 3 min, as otherwise the viability would be reduced. The thawed cells were then transferred into the prepared 15 mL FALCON tube and centrifuged in the centrifuge (Megafuge 1.OR, Kendro Heraeus) at 1,500 rpm for 5 min. The supernatant was then poured off; the resulting pellet was tapped and resuspended with 1 mL of cell culture medium. This suspension was then transferred into the cell skin flask and incubated at 37 °C (water saturated, 5% CO_2_ atmosphere). The cell populations had to be split every 2–3 days on two culture bottles by their growth. The splitting operation corresponds in the beginning to that of the cell counting operation, which is explained next. In the case of splitting, the cell suspension is divided into two culture bottles without the addition of Turks solution. After removal of the frozen cells from the nitrogen tank, the cells were rapidly thawed by immersing the cryocubule (2 mL, Nalgene/NUNC GmbH, Wiesbaden) into a 37.0 °C water bath. Care was taken to limit the defrosting time to a maximum of 3 min since otherwise the viability would be reduced.

### Cell viability and growth

In order to be able to determine the vitality and the growth behavior of the EA.hy 926 cells, the test series were evaluated on the test materials by the AlamarBlue assay and by fluorescence microscopy after calcein acetoxymethyl ester (AM) staining after 24 h of incubation. The metabolic activity of the cells can be measured with the AlamarBlue assay. The tetrazolium salt in AlamarBlue is a redox indicator that changes its fluorospectrometric emission range and color by the cellular metabolic responses. As the number of cells increases, there is an increased reduction in the dye. The nutrient solution is blue at the beginning of the experiment and, depending on the cell activity and the number of cells, the medium becomes increasingly lighter or pink. Only living cells can reduce the dye, whereas by comparing the different approaches with a control, statements can be made about the effect of a test substance or a test material on the cells. First, the PET test materials were placed in a 24-well plate. Coating thicknesses of 100 and 500 nm as well as the two different coating compositions were used. In addition, uncoated material was used as a control to the coatings. A defined cell number of 100,000 cells/well in 1 mL of culture medium was pipetted onto the PET pieces. In addition, two further control wells were determined. A well containing no test material but only cells showed the value for 100% survival rate. The other control well was filled without cells with culture medium and showed the value for no metabolic activity and thus also for 0% survival rate. After an incubation period of 24 h in the incubator at 37 °C (water saturated, 5% CO_2_ atmosphere), the culture medium of the individual wells was carefully pipetted off and to all test well 1 mL of AlamarBlue culture medium solution in the ratio 1:10. Until the color change, which occurred after 3 h, the wells were again incubated in the incubator. In the experiments, the incubation times were maintained during the various test series. On the one hand, the incubation time of 24 h in which the cells adhered to the material and the 3 h in which the AlamarBlue culture medium solution remained in the wells until evaluation began. After the 3 h with the AlamarBlue culture medium solution in which the color change occurred, the 24-well plates were removed from the incubator. Of the now color-altered AlamarBlue culture medium solutions, 1 mL was individually transferred into a new 24-well plate and labeled on the well plate lid according to its origin. The insert was then put into the fluorescence reader. The evaluation was carried out on the fluorescent rectifier FLUOstar OPTIMA (BMG LABTECH, Offenburg, Germany) at 540 nm excitation and 590 nm emission. A total of five series of tests (n=5) were performed with different passages of EA.hy 926 cells. In the case of calcein AM staining, only vital cells are marked. The nonfluorescent calcein AM can penetrate the cell wall and is then cleaved by esterases in the cytosol. This produces calcein, which forms complexes by binding calcium ions. These complexes cannot leave the cell and fluorescence at an excitation of 494 nm and emission of 517 nm. Calcein AM (Calbiochem, Schwalbach, Germany) was first dissolved in 100 μL of DMSO (Sigma-Aldrich, Taufkirchen; Germany) to carry out the staining. This was followed by the addition of 1.1 mL of inactivated FCS and thorough mixing of the solution. The solution was portioned into reaction vessels of 40 μL and stored at −70 °C until use. The staining was carried out after 24 h of incubation time of the cell culture on the sample materials. As controls, a well was used with EA.Hy 926 cells without test material and a well with uncoated dacron material.

### Statistics

The statistical analysis of the results was performed with the program IBM SPSS 23 (IBM, Ehningen, Germany). For the results of fluorescence photometry, the calculation of the relative values compared to the 100% value was done first. For this purpose, first the medium value corresponding to 0% survival rate was subtracted from the values of the respective experimental run and then the relative value was calculated. The arithmetic means as well as the standard deviation and variance were determined for both evaluation methods. A significant difference was assumed at p≤0.05 probability of error.

## Results

### Scanning electron microscopy

**Figure 2:**
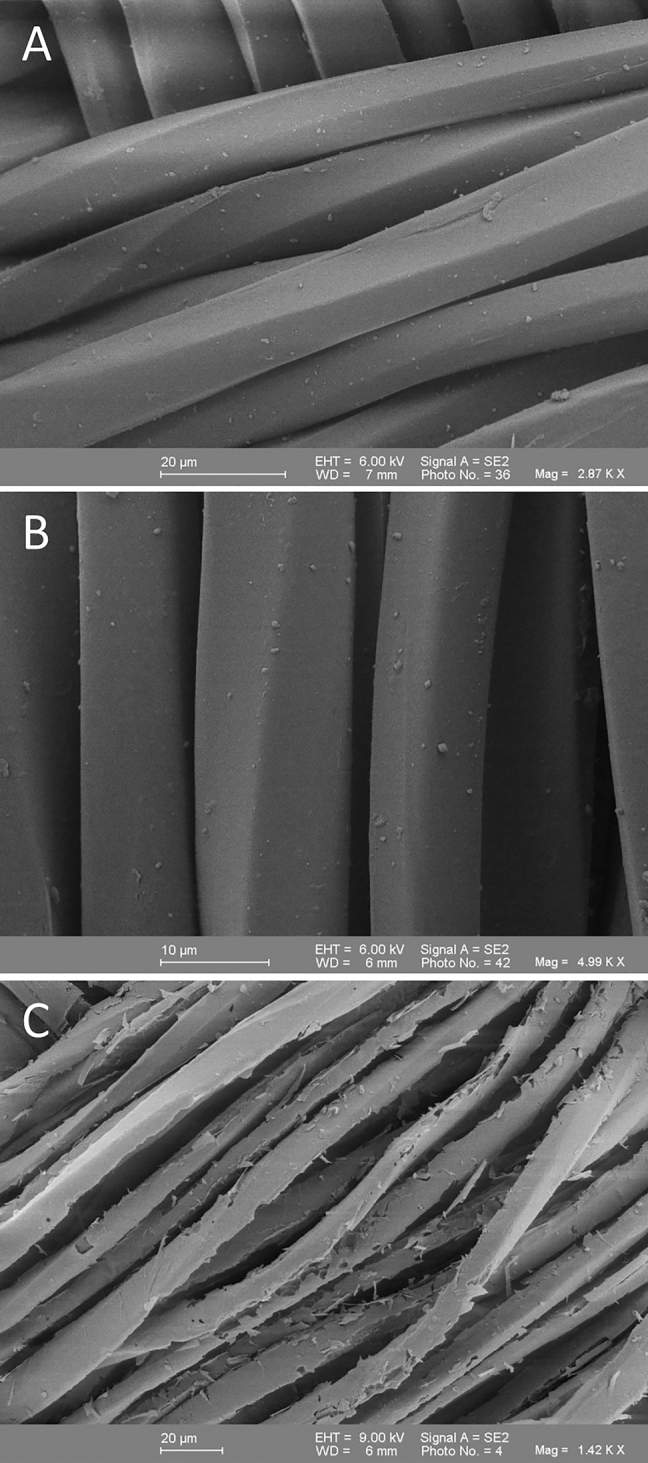
A scanning electron microscope view of a polyethylene terephthalate (PET) vessel prosthesis coated with 100 nm (A), 500 nm (B), and 1,000 nm (C) silicone oxide (200:1). Clearly the peeling of the coating can be seen in (C).

Due to the novelty of the described PET prosthesis coating, they were first examined under the SEM. Firstly, overviews were taken and then details with up to 1,000× magnification could be looked at more closely (Figure 1). At coating thicknesses of 100 and 500 nm, no changes were observed in the surface morphology (Figure 2). The coating thickness of 1,000 nm showed brittle or peeled off areas of the coating (Figure 2C), which is why, the coating thicknesses of 100 nm and 500 nm were only used in the here described test series.

### Fluorescence photometry according to AlamarBlue assay

**Figure 3:**
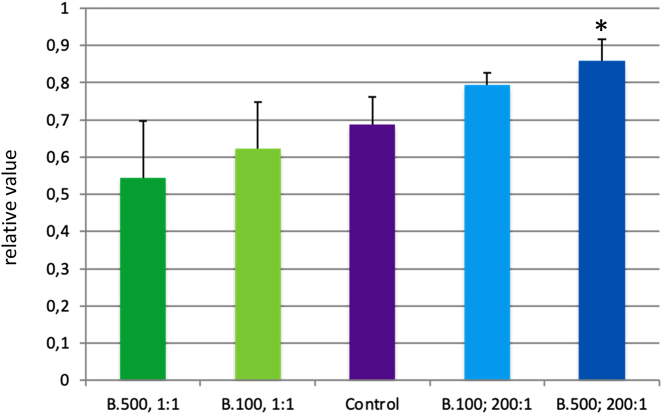
Diagram of the mean ± SD of the activity given by the AlamarBlue assay of the various coatings and the uncoated material (control). Marked with * is the significant difference between uncoated material and coating B.500, 1:1. n=5 per group.

The difference between the coated PET vascular prosthesis material and uncoated material was compared with the substrate conversion of AlamarBlue after 24 h incubation of the EA.hy 926 cells on the material. First, the mean values of the individual samples for the five test series were calculated. In order to detect possible mean value differences, the Levene test was first performed to test variance homogeneity. This showed a significance of p=0.013, which demonstrates an inequality of the variances (p<0.05). Thus, to check possible differences in the mean values of the Games-Howell test, which does not require variance homogeneity compared to untreated PET material, a significantly higher metabolic activity (p=0.026) for the coating 500 nm, ratio 200:1 was shown (Figure 3). The other coatings showed no significant differences compared to the control group with uncoated PET material. Comparing the coatings with each other a significantly lower metabolic activity (p=0.037) of the coating 500 nm, ratio 1:1 compared to the coating 500 nm, ratio 200:1 could be shown (Figure 3). This underlines the apparent tendency of the 1:1 coating to inhibit the metabolic activity of the cells, while the 200:1 coating increases the activity.

### Fluorescence microscopy after calcein AM staining

**Figure 4:**
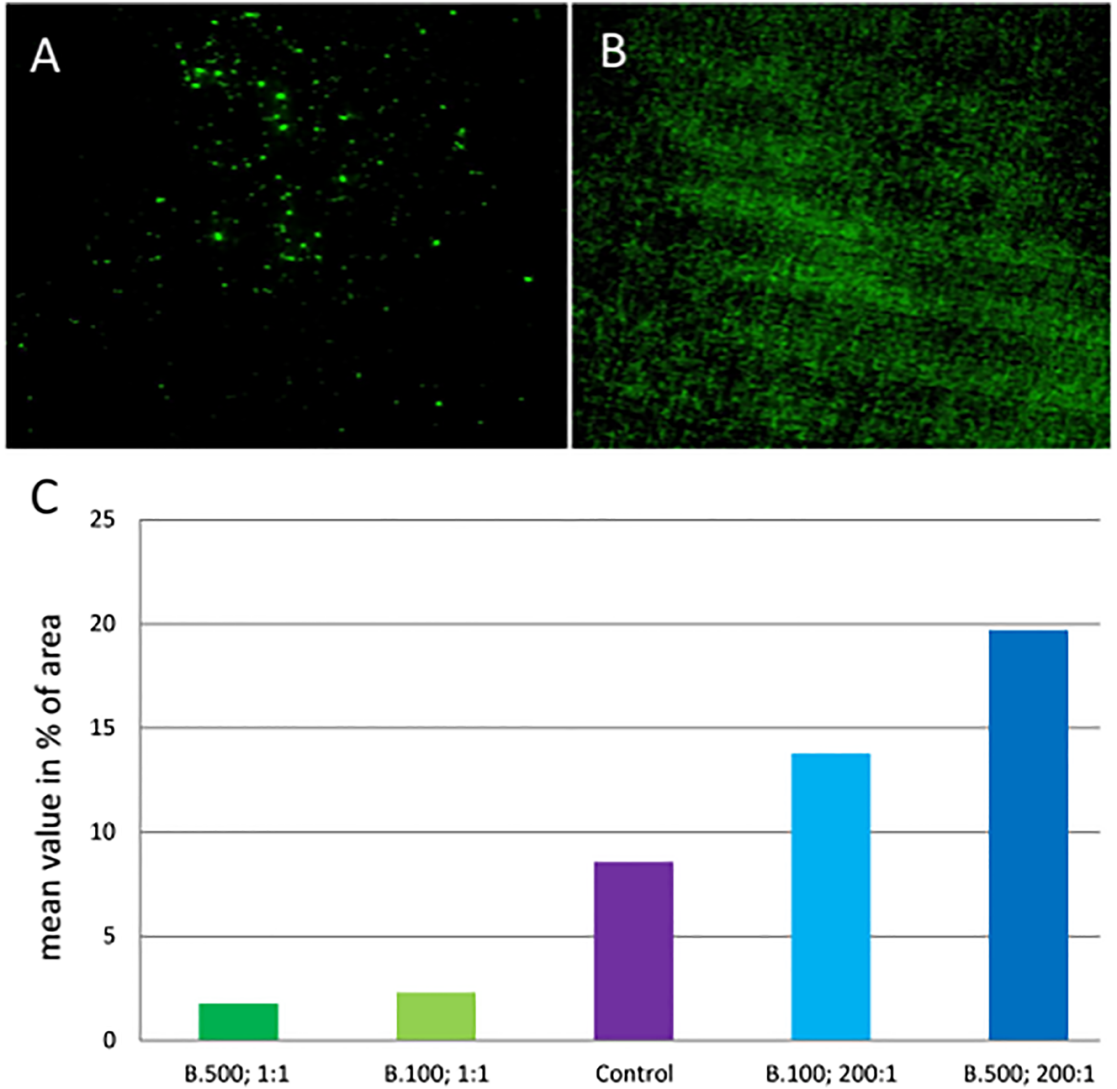
Representative images of the fluorescence uptake of the EA.hy926 cells on polyethylene terephthalate (PET) vessel prosthesis material with the coating 500 nm, 1:1 (A) and with the coating 500 nm, 200:1 (B). Diagram (C) of the mean values as a percentage of the populated total area of the various coatings and the non-coated material (control) after calcein acetoxymethyl ester (AM) staining. n=5 per group.

The difference between the coated PET vascular prosthesis material and the uncoated material was evaluated after 24 h incubation of the EA.hy 926 cells on the material by evaluation of the overgrown percentage area after calcein AM staining. With the help of the Levene test it could be determined again that there was no variance homogeneity of the data (p=0.044). Thus, the Game Howell test was used to investigate possible differences in mean values. In the evaluation of the results of fluorescence microscopy, no significant difference could be detected between the different coatings and the uncoated PET material. However, a tendency of the increased surface growth on the coating 500 nm, ratio 200:1, is shown. The coatings with the ratio 1:1 tend to be less densely covered (Figure 4).

## Discussion

The autologous great saphenous vein bypass is currently superior to an alloplastic vessel replacement and should be used as far as possible [4]. Many different modifications of synthetic (plastic) prostheses have already been developed and investigated for their effects [14]. Surface modifications thus appear to be a possible strategy for developing an alloplastic vascular prosthesis. Intimal hyperplasia is, as already described, a problem in vascular replacement surgery. Since fast endothelialization can lead to the reduction of intimal hyperplasia [15], the development of a coating that promotes endothelialization appears to be suitable [16]. In addition, however, other influencing factors on intimal hyperplasia could also be shown. In vascular prostheses platelet deposition occurs which decreases in the course of time [17]. By reducing surface thrombogenicity, a reduction in intimal hyperplasia has also been demonstrated [18]. Thus, a reduction in surface thrombogenicity displays a further goal of newly developed coatings. The effects of silicone oxide coatings on alloplastic material have so far hardly been investigated. Animal experiments already showed less neointima growth and lower percentages in the stenosis areas using silicone oxide-coated stents [19]. An induction of apoptosis [20, 21] has been shown in studies on exposure to silicon dioxide and quartz dust. Nishimori et al. [22] showed after intravenous injection of silicon oxide nanoparticles in different diameters in mice that there is no liver damage at diameters of 300 nm and 1,000 nm. In contrast, after injection of nanoparticles with a diameter of 70 nm, liver damage was found even at low concentrations. Studies on the biocompatibility of a newly developed coating are therefore sensible and important. As already mentioned, the procedures for vascular prosthesis healing are complex and are subject to various influences. Experiments in cell cultures thus have limited power. However, partial aspects can be examined more closely due to the reduction of complexity. In the present work, the endothelialization of the newly developed silicone oxide-coatings in PET vascular prosthesis material was investigated. The evaluation of the experiments was carried out directly by microscopy after calcein AM staining and indirectly by the evaluation of the AlamarBlue assay.

The AlamarBlue assay was used as an indirect demonstration of the adherence and vitality of the cells. The test is based on the quantification of the metabolic activity of the cells. In this case, however, it cannot be distinguished whether an increased substrate conversion has been achieved by a cell multiplication or an increase in the metabolic activity of the cells. In the evaluation of the results, a significantly higher substrate conversion could be detected in the EA.hy 926 cells on the test material 500 nm, ratio 200:1 compared to the uncoated material. Here, the composition of the coating layer appears to be a significant factor in substrate conversion. For the coating 500 nm, ratio 1:1 a significantly lower metabolic conversion can be shown compared to the coating with a ratio of 200:1. The tendency of the results suggests a poorer metabolic activity in the 1:1 ratio and increased metabolic activity at 200:1 compared to uncoated material (Figure 4). However, these results do not differ significantly, except for those already mentioned. Coloration with calcein AM was necessary since the material is opaque. In fluorescence microscopy, no significant difference in surface colonization could be detected with EA.hy 926 cells. There was only a tendency for better endothelialization in the coating 500 nm with a ratio of 200:1 compared to uncoated material.

In the analysis of the values a variance homogeneity of the data is shown for both test evaluation methods. No significant differences can be observed in fluorescence microscopy. Only a tendency to confirm the results of the AlamarBlue assay is apparent at n=5. To obtain more valid results, an increase in the series of experiments can be useful. In addition to the validity, reliability would also increase.

If one considers the performance of the test evaluation methods (fluorescence microscopy after calcein AM staining and AlamarBlue assay), it is noticeable that the cells were washed several times before fluorescence microscopy. This washing is absent for the AlamarBlue assay and may influence the results. Non-adherent cells were removed during washing. An explanation could also be a metabolic activity of the EA.hy 926 cells enhanced by the silicon oxide coating (200:1). However, this is unlikely with the same tendency of the test evaluation methods and is not documented in the literature so far.

In the work of Muzio et al., mesothelial cells showed an increased production of TGF-β2 in the presence of the aluminum-silicon oxide coating [23]. A direct influence of the surface coating on the metabolic processes and growth of the cells could therefore be present and should be further evaluated. A further explanation for the increased growth on the coating 500 nm, ratio 200:1 could be a change in the surface properties. The change in the surface charge could positively influence the adherence of the endothelial cells. Pavo et al. [19] demonstrated an electrical insulation and filling of metal pores by the silicon on their stents as a positive property, which makes the blood components less adherent to the surface.

Silicone (polydimethylsiloxane [PDMS]) is the most frequently implanted synthetic material in the human body worldwide. At this point, there is also the question to what extent the siliconization of the prosthesis material could lead to negative effects, for example, as described in connection with fibrotic events in the implantation of silicone breast implants or cochlear implants [24, 25]. According to vascular prostheses such problems are not directly known so far. Prosthetic material made of silicone has already been used in vascular surgery for a long time, which did not primarily report the problems that arise, for example, in capsular contracture [26], [27], [28]. On the contrary, even a reduction of intimal hyperplasia using prosthetic material in a combination of PTFE with silicone rubber could be demonstrated [29].

Overall, the coating with silicon oxide with a thickness of 500 nm and the oxygen-HMDSO ratio of 200:1 appears to be a coating which should be further investigated for its effects on endothelial cells. Regarding further factors influencing the vessel prosthesis healing and its problems, further investigations, also in the sense of animal experimental models, are needed to make reliable statements about the influence of the silicon oxide coating on the organism. The development of the intimal hyperplasia as well as the thrombogenicity of the material should be further investigated using histology and immunohistochemistry. In addition, the important physical parameters of permeability, elasticity (compliance), and suturability were to be investigated in flow tests.

## Supplementary Material

Supplementary Material
