# Supplementary material for: Facilitation of adhesion and spreading of endothelial cells on silicone oxide-coated dacron material by microwave-excited low-pressure plasma
Source: Innov Surg Sci. 2022 Jan 14;6(3):97–104. doi: 10.1515/iss-2021-0027 (PMC8826163; doi:10.1515/iss-2021-0027)
Supplement: Supplementary file 1 — Supplementary Material [file j_iss-2021-0027_suppl.pdf]

Reviewer Assessment

H. Sorg et al.: Endothelialization of silicone oxide-coated dacron material by microwave-excited low-pressure plasma

Reviewers' Comments to Original Submission

Reviewer 1: anonymous

Date received: 18-Jun-2021
Reviewer recommendation: **Reject for the following reasons**
Reviewer overall scoring: **Unacceptable**

Assessment Form scores: 5 = High/Yes; 3 = Medium/Adequate; 1 = Low

Is the subject area appropriate for the journal					1
Does the title clearly reflect the paper's content?				2	
Does the abstract clearly reflect the paper's content			3		
Do the keywords clearly reflect the paper's content?			3		
Does the introduction present the problem clearly?			3		
Are the results/ conclusions justified?				2	
How comprehensive and up-to-date is the subject matter presented?				2	
How adequate is the data presentation?					1
Are units and terminology used correctly?			3		
Is the number of cases adequate?					1
Are the experimental methods/ clinical studies adequate?				2	
Is the length appropriate in relation to the content?				2	
Does the reader get new insights from the article?					1
Please rate the practical significance.					1
Please rate the accuracy of methods.					1
Please rate the statistical evaluation and quality control.					1
Please rate the appropriateness of the figures and tables.					1
Please rate the appropriateness of the references.				2	
Please evaluate the writing style and use of language.					1
Please judge the overall scientific quality of the manuscript.					1
Are the methods used worthy of reproduction in greater deal?	No				
Would you be willing to review a revision of this manuscript?	No				

Comments to author: Thank you letting us know of your early experiments; Please state how many did you execute. there is a lot of literature of endothelial cell seeding of vascular grafts; none is cited. More experiments should be performed under in

vitro flow conditions. Please ask a native speaker to do some language editing. Overall I would suggest to submit a revised paper to a journal of bioengineering.

Reviewer 2: anonymous

Date received: 03-Oct-2021
Reviewer recommendation: **Return to author for minor modifications**
Reviewer overall scoring: **High**

Assessment Form scores: 5 = High/Yes; 3 = Medium/Adequate; 1 = Low

Is the subject area appropriate for the journal	5				
Does the title clearly reflect the paper's content?	5				
Does the abstract clearly reflect the paper's content	5				
Do the keywords clearly reflect the paper's content?	5				
Does the introduction present the problem clearly?	5				
Are the results/ conclusions justified?		4			
How comprehensive and up-to-date is the subject matter presented?		4			
How adequate is the data presentation?			3		
Are units and terminology used correctly?		4			
Is the number of cases adequate?			3		
Are the experimental methods/ clinical studies adequate?		4			
Is the length appropriate in relation to the content?		4			
Does the reader get new insights from the article?		4			
Please rate the practical significance.		4			
Please rate the accuracy of methods.	5				
Please rate the statistical evaluation and quality control.		4			
Please rate the appropriateness of the figures and tables.			3		
Please rate the appropriateness of the references.		4			
Please evaluate the writing style and use of language.	5				
Please judge the overall scientific quality of the manuscript.		4			
Are the methods used worthy of reproduction in greater deal?	No				
Would you be willing to review a revision of this manuscript?	Yes				

Comments to author: Dear authors, thank you very much for this well perfomed study, which is nicely presented, both in terms of the content as for the writing. I would thus support its publication, however, would require minor revision concerning the results section. Here, more detailed calculates and numbers are missing (e.g. number of examined specimens, cell growth indicates, etc.); especially, the both figures included look almost identical and would benefit from added values. Lastly, and although you already mentionedthe limitations of the evaluation methods, please comment on how such coating would performe in perfused situations,thus if exposed to the blood flow? would the coating remain stable or peal off?

Reviewer 3: Steinert, Andreas

Date received: 12-Oct-2021
Reviewer recommendation: **Return to author for minor modifications**
Reviewer overall scoring: **High**

Assessment Form scores: 5 = High/Yes; 3 = Medium/Adequate; 1 = Low

Is the subject area appropriate for the journal	5				
Does the title clearly reflect the paper's content?					1
Does the abstract clearly reflect the paper's content		4			
Do the keywords clearly reflect the paper's content?			3		
Does the introduction present the problem clearly?		4			
Are the results/ conclusions justified?		4			
How comprehensive and up-to-date is the subject matter presented?	5				
How adequate is the data presentation?	5				
Are units and terminology used correctly?		4			
Is the number of cases adequate?		4			
Are the experimental methods/ clinical studies adequate?		4			
Is the length appropriate in relation to the content?		4			
Does the reader get new insights from the article?		4			
Please rate the practical significance.		4			
Please rate the accuracy of methods.		4			
Please rate the statistical evaluation and quality control.	5				
Please rate the appropriateness of the figures and tables.		4			
Please rate the appropriateness of the references.	5				
Please evaluate the writing style and use of language.	5				
Please judge the overall scientific quality of the manuscript.		4			
Are the methods used worthy of reproduction in greater deal?	Yes				
Would you be willing to review a revision of this manuscript?	Yes				

Comments to author: Dear Authors, the manuscript presented here, is a new approach to improve the biocompatibility of a commercial vascular prosthesis with a method whose application in the context of the material can be considered as a scientific innovation. This new idea has the potential to reduce the rate of stenosis and occlusion of vascular prostheses in future.
Please note my comments and criticisms:

Title: The title of the manuscript suggests that a method is described, that can induce cellularization of a surface with endothelial cells.
However, the present study merely develops a method to facilitate endothelial cells to adhere to the PET surface (Dacron) and possibly to spread. Therefore, the title needs to be adjusted according to the content and aim of the study.

Keywords: The focus of the manuscript is clearly to augment surface properties of a dacron-vascular-prostehsis- this is your aim and presents the clinical relevance of your study, so it is recommended to choose vascular prosthesis as a key word.

Results: Looking at the graphs, it is noticeable that the values indicating successful coating were achieved by an oxygen-to-HMDSO ratio of 200:1. In your method section (P6,line28) you describe that, by using the oxygen-to-HMDSO ratio of 200:1, it was possible to achieve the lowest possible carbon-coating fraction.
Please explain in your methods section why the lowest possible carbon-coating fraction improves the coating process (P18,Figure3;P19,DiagramC).

Discussion: The Discussion should mention possible disadvantages of surface siliconization, such as fibrotic changes over time (analogous to capsular fibrosis of breast implants), which may have prothrombogenic or mechanical, stenotic properties.

Outlook: Further studies should focus in an in-vivo-model to highlight differences in endothelialization by the organism in cell density, spreading and the associated thrombogenicity using histological sections in an in-vivo-model.
A particular focus should be on rheological blood-flow properties.

Authors’ Response to Reviewer Comments

Date received: 24-Oct-2021

Response to reviewer 1

The authors thank reviewer 1 for the comments and the constructive criticism to improve the content of the paper. We appreciate this and have followed all detailed comments to clarify the manuscript.

Major comments:
1. Thank you letting us know of your early experiments; Please state how many did you execute. there is a lot of literature of endothelial cell seeding of vascular grafts; none is cited. More experiments should be performed under in vitro flow conditions. Please ask a native speaker to do some language editing. Overall I would suggest to submit a revised paper to a journal of bioengineering.

First, we would like to thank the reviewer for his kind comment on our pilot results of this study.
1. number of experiments: We thank the reviewer for his diligent review of the manuscript. The sample number of experiments has now been integrated into the manuscript. For fluorescence photometry according to the AlamarBlue assay we used n=5 per group. For fluorescence microscopy after calcein AM staining we also examined n=5 per group.
We have added and highlighted this accordingly in the materials and methods section and in the figure legends. Please see the revised version of the manuscript marked by red font and underlining.

2. literature of seeding: This is correct. We have added some actual literature on this topic. This part of the introduction now reads as follows: “Various surface coatings, for example, attempt to improve artificial vascular prosthesis material [7-10]. Specifically, the direct and fastest possible cell colonization on the different coatings with endothelial cells was investigated in this context by many groups so far [8, 9, 11-13].” marked by red font and underlining.

3. in vitro flow conditions: This comment from the reviewer is particularly valuable and of interest to the authors as well. We are currently in the planning phase of further, especially in vivo and flow, investigations on this question. What seems also interesting is the possible brittle or peeling effect of the silicone under flowing conditions.
We have emphasized this point even more at the end of the discussion. Here it is now stated as follows: „Overall, the coating with silicon oxide with a thickness of 500 nm and the oxygen-HMDSO ratio of 200: 1 appears to be a coating which should be further investigated for its effects on endothelial cells. Regarding further factors influencing the vessel prosthesis healing and its problems, further investigations, also in the sense of animal experimental models, are needed to make reliable statements about the influence of the silicon oxide coating on the organism. The development of the intimal hyperplasia as well as the thrombogenicity of the material should be further investigated using histology and immunohistochemistry. In addition, the important physical parameters of permeability, elasticity (compliance) and suturability were to be investigated in flow tests.“ marked by red font and underlining.

4. language editing. The manuscript was again revised in some passages by a native speaker. Please see the revised version of the manuscript marked by red font and underlining.

5. bioengineering journal: Thank you very much for this comment. However, we hope that by revising the manuscript we can still convince the reviewer to accept it in Innovative Surgical Science. Especially the open focus in Innovative Surgical Science could make our results accessible to a much broader readership. A positive decision would make the authors very happy.

Response to reviewer 2

The authors thank reviewer 2 for the comments and the constructive criticism to improve the content of the paper. We appreciate this and have followed all detailed comments to clarify the manuscript.

1. Dear authors, thank you very much for this well performed study, which is nicely presented, both in terms of the content as for the writing. I would thus support its publication, however, would require minor revision concerning the results section. Here, more detailed calculates and numbers are missing (e.g. number of examined specimens, cell growth indicates, etc.); especially, the both figures included look almost identical and would benefit from added values. Lastly, and although you already mentioned the limitations of the evaluation methods, please comment on how such coating would perform in perfused situations, thus if exposed to the blood flow? would the coating remain stable or peel off?

First, we would like to thank the reviewer for his kind comment on our study and are pleased that he supports a publication of this manuscript after revision. "

1. sample number/figures: We thank the reviewer for his diligent review of the manuscript. The sample number of experiments has now been integrated into the manuscript. For fluorescence photometry according to the AlamarBlue assay we used n=5 per group. For fluorescence microscopy after calcein AM staining we also examined n=5 per group.

We have added and highlighted this accordingly in the materials and methods section and in the figure legends. Please see the revised version of the manuscript marked by red font and underlining.

We have indeed made a mistake with the figures. The bar charts previously shown in Figures 3 and 4C are incorrectly identical. We have corrected this and have now integrated the correct bar chart in Figures 4C in the revised version. Please see the revised Figure 4.

2. Perfused situations: This comment from the reviewer is particularly valuable and of interest to the authors as well. We are currently in the planning phase of further, especially in vivo, investigations on this question. Especially the stability of the applied silicone coating was of interest to us from the very beginning as well. Thus, we have investigated different thicknesses (100nm, 500nm, 1000nm) within the scope of this project and have already found that the 1000nm coating leads to peeling effects and has therefore not been considered further in the investigation presented here (Figure 2C). However, whether further brittleness or peeling effects occur under flow conditions as prevailing in the human organism is of speculative character at this stage and cannot be answered yet. Therefore, follow-up investigations are mandatory.

We have emphasized this point even more at the end of the discussion. Here it is now stated as follows: „Overall, the coating with silicon oxide with a thickness of 500 nm and the oxygen-HMDSO ratio of 200: 1 appears to be a coating which should be further investigated for its effects on endothelial cells. Regarding further factors influencing the vessel prosthesis healing and its problems, further investigations, also in the sense of animal experimental models, are needed to make reliable statements about the influence of the silicon oxide coating on the organism. The development of the intimal hyperplasia as well as the thrombogenicity of the material should be further investigated using histology and immunohistochemistry. In addition, the important physical parameters of permeability, elasticity (compliance) and suturability were to be investigated in flow tests." marked by red font and underlining.

Response to reviewer 3

The authors thank reviewer 3 for the comments and the constructive criticism to improve the content of the paper. We appreciate this and have followed all detailed comments to clarify the manuscript.

1.Title: The title of the manuscript suggests that a method is described, that can induce cellularization of a surface with endothelial cells. However, the present study merely develops a method to facilitate endothelial cells to adhere to the PET surface (Dacron) and possibly to spread. Therefore, the title needs to be adjusted according to the content and aim of the study.

Thank you for this comment. we have adjusted the title according to the recommendation of the reviewer as follows: "Facilitation of adhesion and spreading of endothelial cells on silicone oxide-coated Dacron material by microwave-excited low-pressure plasma." (see revised version of the manuscript marked by red font and underlining.)

2. Keywords: The focus of the manuscript is clearly to augment surface properties of a dacron-vascular-prosthesis- this is your aim and presents the clinical relevance of your study, so it is recommended to choose vascular prosthesis as a key word.

We have added this key word in the key word section. Please see the revised version of the manuscript marked by red font and underlining.

3. Results: Looking at the graphs, it is noticeable that the values indicating successful coating were achieved by an oxygen-to-HMDSO ratio of 200:1. In your method section (P6,line28) you describe that, by using the oxygen-to-HMDSO ratio of 200:1, it was possible to achieve the lowest possible carbon-coating fraction. Please explain in your methods section why the lowest possible carbon-coating fraction improves the coating process (P18,Figure3;P19,DiagramC).

This is an interesting question raised by the reviewer. It is well known that carbon coating on dacron prostheses facilitates the development of an endothelial monolayer (see for example Bernex et al. and many others). Because we were technically able to adjust the oxygen-to-HMDSO ratio appropriately and could not exclude that this also influenced seeding and spreading of endothelial cells, we examined two different ratios as well. An explanation, as also mentioned in the discussion section, could therefore be an increased metabolic activity of the endothelial cells due to the silicon oxide coating with a ratio of 200:1. However, this cannot be supported by actual literature or other experiences so far. In the work of Muzio et al. (2017), mesothelial cells showed increased production of TGF-ß2 in the presence of the aluminum-silica coating. Accordingly, a direct influence of the surface coating on the metabolic processes and growth of the cells could be present. The increased growth on the 500nm, 200:1 coating could also be explained by be a change in direct surface properties. The change in surface charge could positively influence the adherence of endothelial cells. Pavo et al. (2015) cited electrical isolation and filling of metal pores by the silicon on their stents as a positive property that makes blood components adhere less to the surface. Overall, the 500nm, 200:1 oxygen-HMDSO ratio silicon oxide coating appears to be a coating that should be further investigated for its effects on endothelial cells.

Bernex F, Mazzucotelli JP, Roudiere JL, Benhaiem-Sigaux N, Leandri J, Loisance D. In vitro endothelialization of carbon-coated Dacron vascular grafts. Int J Artif Organs. 1992 Mar;15(3):172-80.

Muzio G, Perero S, Miola M, Oraldi M, Ferraris S, Vernè E, Festa F, Canuto RA, Festa V, Ferraris M. Biocompatibility versus peritoneal mesothelial cells of polypropylene prostheses for hernia repair, coated with a thin silica/silver layer. J Biomed Mater Res B Appl Biomater. 2017 Aug;105(6):1586-1593.

Pavo N, Syeda B, Bernhart A, Szentirmai E, Hemetsberger R, Samaha E, Plass C, Zlabinger K, Pavo IJ, Petrasi Z, Petnehazy Ö, Hoerstrup SP, Maurer G, Gyöngyösi M. Preclinical randomised safety, efficacy and physiologic study of the silicon dioxide inert-coated Axetis and bare metal stent: short-, mid- and long-term outcome. EuroIntervention. 2015 Aug;11(4):433-41.

4. Discussion: The discussion should mention possible disadvantages of surface siliconization, such as fibrotic changes over time (analogous to capsular fibrosis of breast implants), which may have prothrombogenic or mechanical, stenotic properties.

We thank the reviewer for this raised point. Vascular prostheses are regularly made from polyethylene terephthalate (PET, polyester) or polytetrafluorethylene (PTFE). These types of prosthesis materials, however, have evident disadvantages in order of leakage, preclotting or stitch channel bleeding.

Silicone (polydimethylsiloxane, PDMS) is the most frequently implanted synthetic material in the human body worldwide and the implantation of silicone-based implants for breast augmentation is one of the most frequently performed procedures by aesthetic-plastic surgeons. One of the main complications after silicone breast implantation is the development of capsular fibrosis and contracture. The immunologically triggered foreign body reaction (i.e. capsular contracture; CC) leads to functional complications on the implants, which are the cause of the final implant failure. According to vascular prostheses such problems are not known to the authors of this manuscript so far. Prosthetic material made of silicone has already been used in vascular surgery for a long time, which did not primarily report the problems that arise, for example, in capsular contracture (White et al.; Whalen et al.; Stimpson et al.). On the contrary, even a reduction of intimal hyperplasia using prosthetic material in a combination of PTFE with silicone-rubber could be demonstrated (Lumsden et al.).

In addition, there could be a difference in the flowing system, which however would be very interesting to investigate. Interestingly, the fibrosis in airway stenting seems to be an advantageous function using silicon materials in this field (Nesbitt et al.).

We have integrated this point in the discussion section as follows: "Silicone (polydimethylsiloxane, PDMS) is the most frequently implanted synthetic material in the human body worldwide. At this point, there is also the question to what extent the siliconization of the prosthesis material could lead to negative effects, for example, as described in connection with fibrotic events in the implantation of silicone breast implants or cochlear implants (Steiert AE et al.; Kikkawa et al.). According to vascular prostheses such problems are not directly known so far. Prosthetic material made of silicone has already been used in vascular surgery for a long time, which did not primarily report the problems that arise, for example, in capsular contracture (White et al.; Whalen et al.; Stimpson et al.). On the contrary, even a reduction of intimal hyperplasia using prosthetic material in a combination of PTFE with silicone-rubber could be demonstrated (Lumsden et al.)." marked by red font and underlining.

Kikkawa YS, Nakagawa T, Ying L, Tabata Y, Tsubouchi H, Ido A, Ito J. Growth factor-eluting cochlear implant electrode: impact on residual auditory function, insertional trauma, and fibrosis. J Transl Med. 2014 Oct 4;12:280.

Lumsden AB, Chen C, Coyle KA, Ofenloch JC, Wang JH, Yasuda HK, Hanson SR. Nonporous silicone polymer coating of expanded polytetrafluoroethylene grafts reduces graft neointimal hyperplasia in dog and baboon models. J Vasc Surg. 1996 Nov;24(5):825-33.

Nesbitt JC, Carrasco H. Expandable stents. Chest Surg Clin N Am. 1996 May;6(2):305-28.

Steiert AE, Boyce M, Sorg H. Capsular contracture by silicone breast implants: possible causes, biocompatibility, and prophylactic strategies. Med Devices (Auckl). 2013 Dec 2;6:211-8.

Stimpson C, White R, Klein S, Shors E. Patency and durability of small diameter silicone rubber vascular prostheses. Biomater Artif Cells Artif Organs. 1989;17(1):31-43

Whalen RL, Cardona RR, Kantrowitz A. A new, all silicone rubber small vessel prosthesis. ASAIO J. 1992 Jul-Sep;38(3):M207-12.

White RA, Klein SR, Shors EC. Preservation of compliance in a small diameter microporous, silicone rubber vascular prosthesis. J Cardiovasc Surg (Torino). 1987 Sep-Oct;28(5):485-90.

5. Outlook: Further studies should focus in an in-vivo-model to highlight differences in endothelialization by the organism in cell density, spreading and the associated thrombogenicity using histological sections in an in-vivo-model. A particular focus should be on rheological blood-flow properties.

As a result of the promising results of this pilot project, further trials, especially in vivo, are now planned. We have emphasized this point even more at the end of the discussion. Here it is now stated as follows: „Overall, the coating with silicon oxide with a thickness of 500 nm and the oxygen-HMDSO ratio of 200: 1 appears to be a coating which should be further investigated for its effects on endothelial cells. Regarding further factors influencing the vessel prosthesis healing and its problems, further investigations, also in the sense of animal experimental models, are needed to make reliable statements about the influence of the silicon oxide coating on the organism. The development of the intimal hyperplasia as well as the thrombogenicity of the material should be further investigated using histology and immunohistochemistry. In addition, the important physical parameters of permeability, elasticity (compliance) and suturability were to be investigated in flow tests.“ marked by red font and underlining.

Reviewers' Comments to Revised Submission

Reviewer 2: Niesen, Andreas

Date received: 08-Nov-2021
Reviewer recommendation: **Accept in present form**
Reviewer overall scoring: **High**

Assessment Form scores: 5 = High/Yes; 3 = Medium/Adequate; 1 = Low

Is the subject area appropriate for the journal		4			
Does the title clearly reflect the paper's content?		4			
Does the abstract clearly reflect the paper's content		4			
Do the keywords clearly reflect the paper's content?		4			
Does the introduction present the problem clearly?	5				
Are the results/ conclusions justified?			3		
How comprehensive and up-to-date is the subject matter presented?		4			
How adequate is the data presentation?			3		

Are units and terminology used correctly?		4			
Is the number of cases adequate?					
Are the experimental methods/ clinical studies adequate?			3		
Is the length appropriate in relation to the content?		4			
Does the reader get new insights from the article?		4			
Please rate the practical significance.			3		
Please rate the accuracy of methods.		4			
Please rate the statistical evaluation and quality control.			3		
Please rate the appropriateness of the figures and tables.		4			
Please rate the appropriateness of the references.		4			
Please evaluate the writing style and use of language.		4			
Please judge the overall scientific quality of the manuscript.		4			
Are the methods used worthy of reproduction in greater deal?	Yes				
Would you be willing to review a revision of this manuscript?	Yes				

Comments to author: Dear authors, thank you for revising your manuscript according to the comments of the reviewers. By your revision the manuscript significantly improved and gained in clearance. I would now support the acceptance of your work for publication. No further revision is required from my side.

Reviewer 3: Steinert, Andreas

Date received: 27-Oct-2021
Reviewer recommendation: **Accept in present form**
Reviewer overall scoring: **High**

Assessment Form scores: 5 = High/Yes; 3 = Medium/Adequate; 1 = Low

Is the subject area appropriate for the journal	5				
Does the title clearly reflect the paper's content?		4			
Does the abstract clearly reflect the paper's content		4			
Do the keywords clearly reflect the paper's content?	5				
Does the introduction present the problem clearly?		4			
Are the results/ conclusions justified?		4			
How comprehensive and up-to-date is the subject matter presented?	5				
How adequate is the data presentation?	5				
Are units and terminology used correctly?		4			
Is the number of cases adequate?		4			
Are the experimental methods/ clinical studies adequate?		4			
Is the length appropriate in relation to the content?		4			
Does the reader get new insights from the article?		4			
Please rate the practical significance.		4			
Please rate the accuracy of methods.		4			

Please rate the statistical evaluation and quality control.	5				
Please rate the appropriateness of the figures and tables.		4			
Please rate the appropriateness of the references.	5				
Please evaluate the writing style and use of language.	5				
Please judge the overall scientific quality of the manuscript.		4			
Are the methods used worthy of reproduction in greater deal?					
Would you be willing to review a revision of this manuscript?	No				

Comments to author: Thank you for editing the manuscript according to my Review.

I now have recommended the publication of your corrected version of the manuscript.
